# Seroprevalence of brucellosis among high-risk individuals in Madinah, Saudi Arabia

**DOI:** 10.14202/vetworld.2024.1661-1666

**Published:** 2024-08-03

**Authors:** Mustafa A. Najim, Yahya A. Almutawif, Hamza M. A. Eid, Amjad M. Yousuf, Hazem A. Alahmadi, Muath E. Alharbi, Ziad O Aljabri, Hatim M. Makhdoom, Mohammed S. Yoniss, Ibrahim H.A. Abd El-Rahim, Amer Alshengeti

**Affiliations:** 1 Department of Clinical Laboratories Sciences, College of Applied Medical Sciences, Taibah University, Madinah 41411, Saudi Arabia; 2 Department of Environmental and Health Research, Umm Al-Qura University, Makkah 21955, Saudi Arabia; 3 Department of Pediatrics, College of Medicine, Taibah University, Al-Madinah 41491, Saudi Arabia; 4 Department of Infection Prevention and Control, Prince Mohammad Bin Abdulaziz Hospital, National Guard Health Affairs, Al-Madinah 41491, Saudi Arabia

**Keywords:** *Brucella* IgG, *Brucella* IgM, brucellosis, Madinah, serological assays, undulant fever, zoonosis

## Abstract

**Background and Aim:**

Brucellosis is a highly contagious, neglected zoonotic disease of major importance worldwide. The disease is endemic in many countries, burdening healthcare systems and the livestock industry and representing a persistent public health concern in these countries. Brucellosis is considered an important occupational hazard for livestock workers. Limited studies have investigated human brucellosis in Saudi Arabia. Therefore, this study aimed to estimate the prevalence of brucellosis among employees of high-risk brucellosis professions, including veterinarians, animal herders, and abattoir workers in Madinah, Saudi Arabia, and to determine the associated risk factors.

**Materials and Methods:**

A cross-sectional study was conducted in Madinah, Saudi Arabia, during the period of January–March 2023. Ninety blood samples were collected from individuals occupationally at risk of exposure to *Brucella* infections. Serum samples were examined for immunoglobulins (Ig)M and IgG antibodies against *Brucella* using an indirect enzyme-linked immunosorbent assay. Before sample collection, a predesigned online questionnaire was used to collect the participants’ sociodemographic characteristics and the probable risk factors for human brucellosis. A Chi-square test was used to compare the differences among groups; p *<* 0.05 were considered statistically significant.

**Results:**

Among the 90 participants among the high-risk individuals, *Brucella* IgM and IgG seropositivity were found in 8 (8.8%) and 11 (12.12%) cases, respectively. IgM mono antibody positivity was observed in 4 (4.44%) and 7 (7.77%) of the study population who tested positive for IgG only. Dual positivity for IgM and IgG antibodies was observed in 4 (4.44%) participants. No significant association was determined between seropositivity and age, urbanicity, education, occupation, and duration of exposure (p > 0.05).

**Conclusion:**

Brucellosis is a high-risk occupational disease among workers with close contact with livestock. This study demonstrates that the seroprevalence of brucellosis among occupationally high-risk individuals in Madinah, Saudi Arabia, is relatively low compared to other countries in the region. Nevertheless, educational programs should be implemented to improve knowledge regarding brucellosis, particularly among high-risk individuals.

## Introduction

Brucellosis is a highly contagious, neglected zoonotic disease that is endemic in many low- and middle-income countries, placing a burden on health-care systems and the livestock industry, representing a persistent global health issue [1, 2]. The disease is also known as Malta fever, Mediterranean fever, and undulant fever. It is caused by Gram-negative bacteria of the genus *Brucella*, facultative intracellular bacteria [3, 4]. There are multiple species of *Brucella*, including *Brucella abortus*, *Brucella melitensis*, *Brucella suis*, *Brucella canis*, and *Brucella ovis*. Importantly, *B. melitensis*, *B. abortus*, and *B. suis* are the leading causes of human brucellosis [5, 6]. Brucellosis transmission to humans occurs mainly through contact with infected animals and consumption of raw dairy products or undercooked meat [7, 8]. Human-to-human transmission of brucellosis is rare, but it is possible through various routes, including the transplacental route, breastfeeding, sexual intercourse, blood transfusion, and bone marrow transplantation [9]. Human brucellosis may result in acute or chronic illnesses characterized by non-specific symptoms, including undulant fever, headache, fatigue, backache, joint pain, muscle pain, and night sweating [10]. Globally, 500,000 cases of human brucellosis are reported annually. However, the correct incidence was estimated to be between 5,000,000 and 12,500,000 cases per year [11]. Although numerous developed countries, including Australia, Canada, Japan, and New Zealand, have eradicated the disease, brucellosis remains a public health issue in Africa, the Middle East, parts of Asia, and Latin America as a result of its high endemicity in these regions [12]. In Saudi Arabia, the Department of Agriculture mandated *Brucella* vaccination for livestock and imported animals, which resulted in a reduction in incidence rates. However, the country still considers the disease endemic [13]. The incidence of the disease in the country remains high compared to the world average, it has been estimated to be 15.34/100,000 annually from 2003 to 2018 [4]. The seroprevalence of brucellosis in the country varies among different regions; it was reported at 8.6% in the Wadi Al Dawaser region and 16% and 19% in the Southwestern and southern regions, respectively [14].

A previous seroprevalence report showed a national seroprevalence of 15% among the Saudi population [15]. Veterinarians, animal herders, and slaughterhouse workers are at higher risk of contracting *Brucella* infection due to their frequent direct contact with animals [16]. Infectious disease surveillance is an essential part of public health practices that involves monitoring the burden and epidemiology of the disease. This will help determine the transmission parameters of the disease, thereby helping apply prevention and control measures. Few studies have evaluated the prevalence of human brucellosis in the region, and no study targeted high-risk individuals in the Madinah region.

This study aimed to estimate the prevalence of brucellosis among high-risk occupational groups in Madinah, Saudi Arabia, by determining anti-*Brucella* immunoglobulin (Ig)M and IgG antibodies using enzyme-linked immunosorbent assay (ELISA).

## Materials and Methods

### Ethical approval and informed consent

This study was approved by Research Ethics Committee, College of Applied Medical Sciences, Taibah University, with approval number 2023/145/102 MLT. All participants were informed that participation in the study is voluntary and restricted to employees of high-risk brucellosis professions. A prestructured online questionnaire was designed to collect data on sociodemographic characteristics, including age, urbanicity, occupation, education, and occupation period. Before questionnaire administration, verbal informed consent was obtained from each eligible participant.

### Study period and location

This cross-sectional study was conducted from January to March 2023 in Madinah, Saudi Arabia.

### Sample collection

In total, 90 blood samples were randomly collected from high-risk individuals in Madinah. A trained health professional used a sterile disposable syringe to collect 5 mL of blood from each participant through the brachial vein. The collected samples were then transferred into pre-labeled plain vacutainer tubes. The samples were then centrifuged at 3000× *g* for 10 min, and the separated serums were transferred into new labeled tubes and stored at –20°C until use.

### Serological investigation

Serum samples were screened for human antibodies against *Brucella* lipopolysaccharide using indirect ELISA. The commercial ELISA kits were obtained from Vircell, Spain, with catalog numbers M1006 and G1003 for IgM and IgG, respectively. The tests were performed according to the manufacturer’s instructions, and the samples’ optical density (OD) was recorded at 450/620 nm using an ELISA microwell plate reader. The validity of the kits and assays was confirmed based on the range of positive control, negative control, and the cutoff control provided by the manufacturer. Then, results were interpreted as positive or negative based on the antibody index of each sample, which was calculated using the OD of cutoff control and the OD of the sample. The index value was considered positive above 11, indeterminate between 9 and 11, and negative below 9. According to the manufacturer, the sensitivity and specificity of *Brucella* ELISA IgM were 89% and 100%, respectively, and for *Brucella* ELISA IgG were 98% and 100%, respectively.

### Statistical analysis

The laboratory results were entered into Microsoft Excel 2016 spreadsheets (Microsoft, Washington, USA) containing demographic data generated from an online questionnaire. Statistical analysis was performed using GraphPad Prism 9 software (San Diego, California, USA). Percentages and frequencies were calculated using descriptive analysis and the Chi-square test (χ^2^) was used to determine the association between prevalence of *Brucella* antibodies and categorical variables. p < 0.05 was considered statistically significant.

## Results

### Baseline characteristics of the participants

A total of 90 individuals from occupationally high-risk groups with brucellosis were recruited throughout different regions across Madinah City. The age group distribution of the participants showed a larger proportion of 31–40 years old group (47.78%), followed by 41–50 years old (22.22%), 20–30 years old (18.89%), and more than 50 years old group represented only (11.11%). In this study, majority of the participants (84.44%) were residents of rural areas, while a minority of them lived in urban areas (15.56%). Regarding the educational level of the study subjects, 32.22% have primary education, while 31.11% have undergraduate level and 23.33% of them have a secondary level of education, the minority of them have no education (13.3%). Out of the 90 participants, 65.56% were herders of either cows, camels, or sheep. Abattoir workers and veterinarians represented 24.44% and 10%, respectively. Finally, regarding the occupation period, less than half of the study subjects (41.11%) had worked for 0–5 years, while 37.78% of them had worked for more than 10 years, and the rest 21.11% of them spent 6–10 years (Table-1).

**Table-1:** *Brucella* IgM seroprevalence in study subjects by sociodemographic characteristics and results of variable analysis.

Variable	Category	Frequency (%)	IgM				Chi- square	p-value

			Negative	Positive	% Within +ve IgM	Prevalence (%)		
Age	20–30	17 (18.89)	14	3	37.5	17.65	2.04	0.563
	31–40	43 (47.78)	40	3	37.5	6.98		
	41–50	29 (32.22)	27	2	25	6.9		
	51 and more	1 (1.11)	1	0	0	0		
Residency	Rural	76 (84.44)	68	8	100	10.53	1.62	0.203
	Urban	14 (15.56)	14	0	0	0		
Education	None	12 (13.33)	11	1	12.5	8.33	1.81	0.612
	Primary	29 (32.22)	26	3	37.5	10.34		
	Secondary	21 (23.33)	18	3	37.5	14.29		
	Graduate and above	28 (31.11)	27	1	12.5	3.57		
Occupation	Herder	59 (65.56)	52	7	87.5	11.86	2.04	0.361
	Abattoir worker	22 (24.44)	21	1	12.5	4.55		
	Veterinarian	9 (10)	9	0	0	0		
Duration of Exposure	0–5 Years	37 (41.11)	33	4	50	10.81	0.48	0.788
	6–10 Years	19 (21.11)	18	1	12.5	5.26		
	10 years and more	34 (37.78)	31	3	37.5	8.82		

Ig=Immunoglobulin

### Characteristics of *Brucella* IgM seropositivity among the study population

The overall seroprevalence of anti-*Brucella* IgM antibodies was 8.8% (n = 8) among the 90 healthy participants. This study found IgM seropositivity ranged from 17.65% among age group of 20–30 years (n = 17) to 6.98% among age group of 31–40 (n = 43), followed by 6.9% among 41–50 (n = 29) and 0% was recorded in age groups of 51 and more (n = 1), but the difference in IgM rates among these age groups did not reach statistical significance (p > 0.05). Regarding residency, IgM seropositivity was higher in rural residents, 10.53% (n = 76), compared to 0% (n = 14) among those who live in urban areas. However, this difference was not statistically significant (p > 0.05). Based on the educational level, no significant difference was observed in IgM seropositivity among participants with no education, primary education, secondary education, and graduate and above (p > 0.05). According to the occupations of the participants, none of the 9 veterinarians were found to be seropositive 0% (n = 9), while cattle herders and abattoir workers had higher IgM seropositivity 11.86% (n = 59) and 4.55% (n = 22), respectively. However, this difference was not statistically significant (p > 0.05). Furthermore, IgM seropositivity among the participants was not affected by the duration of exposure; it was 10.81% (n = 37), 5.26% (n = 19), and 8.82% (n = 34) among those who had worked for 0–5 years, 6–10 years, and those who had worked 10 years and more, respectively (Table-1).

### Characteristics of *Brucella* IgG seropositivity among the study population

Among 90 participants, the seropositivity for anti-*Brucella* IgG antibodies was 12.12% (n = 11). This seropositivity was stratified by age, urbanicity, educational level, occupation, and duration of exposure (Table-2). Looking at the age groups, the highest IgG rate was recorded among participants aged between 20 and 30 years old 23.53% (n =17), followed by the 41–50 years of age group 17.24% (n = 29) and the 31–40 years of age group (4.65%) (n = 43). In contrast, no seropositivity was recorded in 51 years and more (0%) (n = 1), statistical analysis revealed no significant difference among these age categories (p > 0.05). Regarding urbanicity, a high prevalence of IgG was reported among rural residents, 13.16% (n = 10), compared to urban residents, 7.14% (n = 14). However, this difference was not statistically significant (p > 0.05). The prevalence of IgG antibodies was also evaluated on the basis of education level; no significant difference was reported among participants of different educational levels (p > 0.05). According to occupation, *Brucella* IgG was higher among herders of animals, in comparison to abattoir workers and veterinarians. However, this difference was not statistically significant (p > 0.05). Regarding the duration of exposure of the study subjects, IgG seropositivity ranged from 17.65% (n = 34) among those who had worked for 10 years in occupation, followed by 13.51% (n = 37) among those who had worked for 0–5 years to and 0% (n = 19) among those had worked for 6–10 years. However, this difference was not statistically significant (p > 0.05) (Table-2).

**Table-2:** *Brucella* IgG seroprevalence in study subjects by sociodemographic characteristics and results of variable analysis.

Variable	Category	Frequency (%)	IgG				Chi- square	p-value

			Negative	Positive	% within +ve IgG	Prevalence (%)		
Age	20–30	17 (18.89)	13	4	36.36	23.53	5.14	0.162
	31–40	43 (47.78)	41	2	18.18	4.65		
	41–50	29 (32.22)	24	5	45.45	17.24		
	51 and more	1 (1.11)	1	0	0	0		
Residency	Rural	76 (84.44)	66	10	90.91	13.16	0.4	0.528
	Urban	14 (15.56)	13	1	9.09	7.14		
Education	None	12 (13.33)	10	2	18.18	16.67	1.04	0.791
	Primary	29 (32.22)	25	4	36.36	13.79		
	Secondary	21 (23.33)	18	3	27.27	14.29		
	Graduate and above	28 (31.11)	26	2	18.18	7.14		
Occupation	Herder	59 (65.56)	49	10	90.91	16.95	3.69	0.158
	Abattoir worker	22 (24.44)	21	1	9.09	4.55		
	Veterinarian	9 (10)	9	0	0	0		
Duration of exposure	0–5 years	37 (41.11)	32	5	45.45	13.51	3.64	0.162
	6–10 years	19 (21.11)	19	0	0	0		
	10 years and more	34 (37.78)	28	6	54.55	17.65		

Ig=Immunoglobulin

### Frequency of anti-*Brucella* antibodies of study subjects

**Figure-1:**
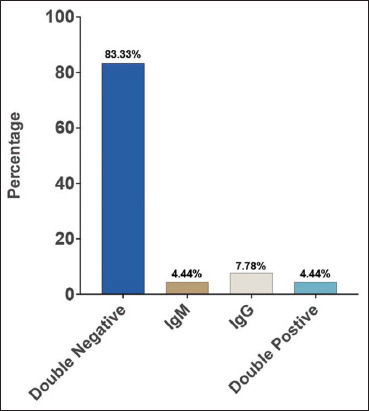
Prevalence of anti-brucella IgM and IgG antibodies among the 90 study participants. Ig=Immunoglobulin.

The prevalence of detecting anti-*Brucella* antibodies was 8.8% (n = 8) by IgM and 12.12% (n = 11) by IgG. IgM mono antibody positivity was observed in 4 (4.44%) and 7 (7.77%) of the study population tested positive for IgG only (Figure-1). On the other hand, there were 4 (4.44%) participants with dual positivity for both IgM and IgG antibodies.

## Discussion

This study aimed to estimate brucellosis seroprevalence as an occupational zoonotic disease among practicing veterinarians, animal herders, and abattoir workers, and the associated risk factors. In Saudi Arabia, the reported cases of *Brucella* were higher than the global average [4]. This was attributed to the fact that Saudi Arabia is one of the highest animal importing countries, as it needs to meet the local demands of red meats throughout the year and slaughterhouses during the Hajj and Umrah seasons [4]. Brucellosis is a high-risk occupational zoonotic disease among veterinarians, animal herders, and abattoir workers, particularly in brucellosis-endemic countries. Ninety blood samples were collected from occupationally high-risk individuals of brucellosis from different regions in Madinah City. This seroprevalence was performed using commercial IgM and IgG ELISA assays; this assay was recommended to be chosen for detecting brucellosis among suspected cases in clinical practice due to its high sensitivity and specificity [17, 18].

The seroprevalence rate of *Brucella* antibodies among the targeted population was 12.12% and 8.8% for IgG and IgM, respectively. IgM seropositivity rate (8.8%) in our study was relatively similar to the reported rate of 9.2% among high-risk groups in Bangladesh [19]. IgM rate in our study was higher than 6.1% reported in Bauchi State, Nigeria [20], and 2.1% among rural Pastoralist communities in Kenya [21]. In contrast, a seroprevalence study reported no IgM positivity among 75 occupationally high-risk individuals in Western Rajasthan, India [22]. Importantly, the detection of these IgM antibodies among study subjects is indicative of (early) active infection, suggesting local transmission of *Brucella* among the targeted community and confirming the disease endemicity in the targeted area of the country. Altogether, our study reveals the presence of human brucellosis in occupationally exposed individuals, specifically animal herders and abattoir workers in the Madinah region. Furthermore, this study showed no significant difference in the distribution of *Brucella* IgM antibodies between different variables, including age, urbanicity, education, occupation, and duration of exposure. Unfortunately, these findings could be explained by the low sample size of the study due to difficulties experienced in the recruitment of study participants.

On the other hand, this study reveals that the brucellosis IgG seroprevalence rate is comparable to reported rates in other countries. Our finding is similar to a study performed in Turkey that reported an 11.8% prevalence of occupational brucellosis among veterinary personnel [23]. In addition, our study revealed relatively higher seropositivity compared to 13.3% reported among veterinarians and slaughterhouse workers in Hamadan, Western Iran [24]. A seroprevalence study using indirect ELISA showed a relatively higher rate of *Brucella* antibodies, 15.69%, among high-risk group individuals working in India [17]. In contrast, a higher prevalence rate was reported among veterinarians in neighboring countries. For example, 43.9% and 76% were reported in Jordan [25] and Northern Palestine [26], respectively. Comparatively, in an Egyptian study, brucellosis seropositivity prevalence was 57.3% among occupationally exposed workers [27]. Moreover, there was no significant difference in the distribution of *Brucella* IgG antibodies among different characteristics of study subjects, including age, residency, education, occupation, and duration of exposure. Our result suggests further comprehensive epidemiological studies for human brucellosis are recommended to clearly estimate the epidemiological aspects of the disease among high-risk individuals in Saudi Arabia. This will clearly contribute to determining the disease burden and identifying its associated risk factors in the country. Moreover, it will contribute to the strategic planning of infection prevention and control programs. This study has demonstrated that brucellosis is an important public health concern among occupationally exposed workers in Madinah. Brucellosis is preventable through adherence to proper work hygiene and safe practices, including good general hygiene, wearing personal protective devices during contact and slaughtering of animals, and providing appropriate medical care to the infected workers [20]. Education campaigns are encouraged to increase awareness about brucellosis, especially among those with a high-risk of contracting the infection.

### Limitations

Our study had some limitations. First, our study was constrained by a relatively small sample size as we could not establish the association between *Brucella* antibodies and different factors of study subjects. This aspect will be considered during the further development of the project. Second, it is worth noting that our research did not evaluate animal brucellosis within the targeted region. Addressing such a gap would provide a more comprehensive picture of the disease transmission and overall impact on the city. Notwithstanding, these earlier limitations, to the best of our knowledge, no prior study investigated the prevalence of *Brucella* antibodies among high-risk occupational individuals in Madinah. A notable strength of the study lies in its measurement of both IgG and IgM rates, as relying solely on IgG could result in the oversight of cases during the active phase of infection.

## Conclusion

The small-scale survey of occupationally high-risk individuals in Madinah, Saudi Arabia, reveals a relatively low seroprevalence of brucellosis compared to other countries in the region. In Madinah, Saudi Arabia, regular contact with livestock increases the risk of contracting the disease and confirms its endemic presence and local transmission among workers. To minimize *Brucella* exposure in high-risk groups, community and worker awareness of the disease and its risk factors should be heightened. Screening for brucellosis in humans and animals, as well as treatment for infected workers, is imperative. Our findings provide a foundation for creating intervention strategies to halt the transmission of the disease in the study area.

## Authors’ Contributions

MAN, YAA, HMAE, AMY, HMM, AA: Designed and planned the study protocol, drafted the manuscript, and participated in data analysis. HAA, MEA, ZOA, MSY, IHAAE: Contributed to data and sample collection and ELISA work. All authors have read, reviewed, and approved the final manuscript.
